# Exploring secondary SARS-CoV-2 transmission from asymptomatic cases using contact tracing data

**DOI:** 10.1186/s12976-021-00144-z

**Published:** 2021-07-16

**Authors:** Ko Nakajo, Hiroshi Nishiura

**Affiliations:** 1 Graduate, School of Medicine, Hokkaido University, Kita 15 Jo Nishi 7 Chome, Kita-ku, Sapporo-shi, Hokkaido, 060-8638 Japan; 2 Kyoto University School of Public Health, Yoshidakonoecho, Sakyo-ku, Kyoto-shi, Kyoto, 606-8503 Japan

**Keywords:** Asymptomatic case, Reproduction number, Cluster, Epidemiology, Mathematical model

## Abstract

**Background:**

Individuals with asymptomatic severe acute respiratory syndrome coronavirus-2 (SARS-CoV-2) infection can propagate the virus unknowingly and thus have been a focus of public health attentions since the early stages of the pandemic. Understanding viral transmissibility among asymptomatic individuals is critical for successful control of coronavirus disease 2019 (COVID-19). The present study aimed to understand SARS-CoV-2 transmissibility among young asymptomatic individuals and to assess whether symptomatology was associated with transmission of symptomatic vs. asymptomatic infections.

**Methods:**

We analyzed one of the first-identified clusters of SARS-CoV-2 infections with multiple chains of transmission that occurred among university students in March 2020 in Kyoto prefecture, Japan, using discrete and two-type branching process models. Assuming that the number of secondary cases resulting from either primary symptomatic or asymptomatic cases independently followed negative binomial distributions, we estimated the relative reproduction numbers of an asymptomatic case compared with a symptomatic case. To explore the potential association between symptomatology and transmission of symptomatic vs. asymptomatic incident infections, we also estimated the proportion of secondary symptomatic cases produced by primary symptomatic and asymptomatic cases.

**Results:**

The reproduction number for a symptomatic primary case was estimated at 1.14 (95% confidence interval [CI]: 0.61–2.09). The relative reproduction number for asymptomatic cases was estimated at 0.19 (95% CI: 0.03–0.66), indicating that asymptomatic primary cases did not result in sufficient numbers of secondary infections to maintain chains of transmission. There was no apparent tendency for symptomatic primary cases to preferentially produce symptomatic secondary cases.

**Conclusions:**

Using data from a transmission network during the early epidemic in Japan, we successfully estimated the relative transmissibility of asymptomatic cases of SARS-CoV-2 infection at 0.22. These results suggest that contract tracing focusing on symptomatic index cases may be justified given limited testing capacity.

**Supplementary Information:**

The online version contains supplementary material available at 10.1186/s12976-021-00144-z.

## Background

Coronavirus disease 2019 (COVID-19) is an infectious disease caused by severe acute respiratory syndrome coronavirus 2 (SARS-CoV-2) that reached pandemic levels in 2020. Clinical manifestations range from non-specific upper or lower respiratory symptoms to severe pneumonia and death. The case fatality risk depends on age: the elderly are the most vulnerable group with approximately 300 deaths occurring per 1000 patients aged 85 years or older [1]. Vaccination of targeted groups including the elderly has just begun in some countries, but it remains unclear whether the pandemic will come to an end in the near future.

Individuals with asymptomatic SARS-CoV-2 infection (i.e., individuals who never develop symptoms throughout the course of infection) have been a focus of public health attentions since the early stages of the pandemic [2–4]. Because these individuals can propagate the virus unknowingly, elucidating the transmissibility of asymptomatic infections is critical for successful control of COVID-19. If asymptomatic infections are frequent and transmissibility is substantial, controlling the epidemic via screening of symptomatic cases might not be an effective strategy. However, if the transmissibility of asymptomatic cases is limited, health authorities can allocate limited resources to tracing primary symptomatic cases to bring the epidemic under control [5–7].

The transmissibility of asymptomatic infections remains unclear [7–9]. The results of several studies have been contradictory [8, 10–16]. In a retrospective study of 303 symptomatic and asymptomatic patients in a community treatment center in Korea, the viral loads of asymptomatic patients were similar to those of symptomatic patients [10]. Other studies leveraging viral load as a surrogate of transmissibility supported this notion [11, 12]. Two epidemiological studies conducted in Singapore and Brunei showed that the incidence rate ratio (asymptomatic vs. symptomatic cases) and attack rate ratio (asymptomatic and pre-symptomatic vs. symptomatic cases) were both below 1 (0.24 and 0.78, respectively), suggesting that asymptomatic infections may be less transmissible than symptomatic infections [13, 14]. He et al. [15] analyzed transmission data in Ningbo from January 21 to March 6, 2020 [17] and estimated the reproduction numbers of asymptomatic and symptomatic cases as 0.20 and 0.78, respectively. This finding indicated that the relative transmissibility of asymptomatic cases was below 1, and was further supported by the work of Nakajo and Nishiura in analyzing transmission trees among older adults [16].

These findings need to be strengthened and extended to other populations and age groups (e.g., young adults). Moreover, it remains unclear whether transmission from asymptomatic cases is more likely to lead to asymptomatic infections. Here we analyzed a cluster of SARS-CoV-2 infections in Japan that were propagated mainly among university students. We aimed to understand the transmissibility of asymptomatic SARS-CoV-2 infections among young adults and examine the potential role of symptomatology in giving rise to secondary symptomatic vs. asymptomatic infections.

## Materials and methods

### Epidemiological data

**Fig. 1:**
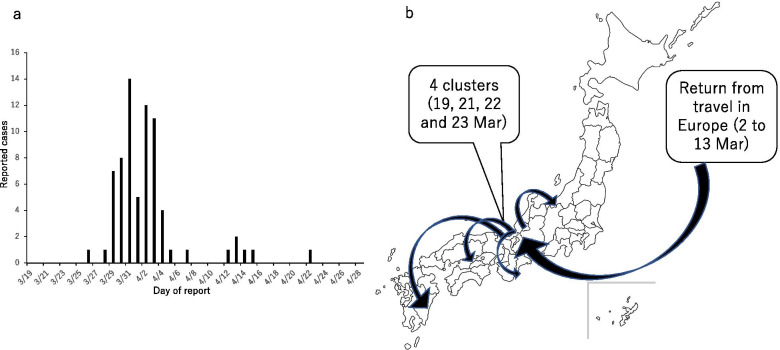
Epidemiological characteristics in a cluster of SARS-CoV-2 infections among university students in Kyoto, Japan. **a** Epidemic curve. Daily counts of confirmed cases are shown as a function of the day of report. The recognition of the cluster was notified on 29 March 2020. **b** Spatial propagation of the cluster. The three index cases were university students returning from travel in Europe. Four clusters were identified in Kyoto and secondary or additional cases were reported across the country

We analyzed one of the first-identified clusters of SARS-CoV-2 infections among university students with multiple chains of transmission. The cluster occurred in Kyoto prefecture, Japan, in March 2020 and involved a total of 74 confirmed cases. The three index cases traveled to Europe in early March to celebrate their graduation from university, returning to Japan on 14 March 2020. Subsequently, transmission events took place during three parties on the nights of 19, 21, and 22 March in Kyoto, which were attended by the index cases independently. One of the secondary cases infected at one of the parties contributed to subsequent transmission events during another party on 23 March. The first confirmed case, which later turned out to be one of the index cases, was reported on 26 March in Ehime prefecture, southwest of Kyoto. Additional cases were notified in other prefectures as well as in Kyoto, forcing local public health centers to start contact tracing on 29 March (Fig. 1a, b). The original data used to construct the epidemic curve are available as Online Supporting Material.

Cluster-based approaches in Japan identified indoor environments as focal areas of transmission. All close contacts of confirmed cases that could be identified retrospectively were brought under observation and subjected to laboratory testing [18, 19]. Accordingly, clusters from January to March 2020 were extremely well traced, and importantly, incidence did not exceed contact tracing capacity. Movement of all identified close contacts was restricted for 14 days and they underwent laboratory testing by polymerase chain reaction to confirm SARS-CoV-2 infection. Information on transmission networks, age, symptomatic status (i.e., manifested any symptoms by the end of isolation vs. never manifested symptoms) and time of illness onset (for symptomatic cases only) was collected.

### Mathematical model

We explored SARS-CoV-2 transmissibility and its dependence on symptomatology using discrete and two-type branching process models. Offspring distributions of SARS-CoV-2 infections, characterized by superspreading events, have conventionally been modeled using the negative binomial distribution [20–22]. We adhered to this custom, assuming that the number of secondary cases arising from either primary symptomatic or asymptomatic cases independently followed negative binomial distributions with means $R_{s}$ or $R_{a}$, respectively, and common dispersions $k$. We set $v$ as the relative reproduction number of asymptomatic cases compared with symptomatic cases such that $R_{a} = vR_{s}$. Let $D$ represent observed data (with total sample size *n*), then we can describe the likelihood of observing the number of secondary cases as:

1 $$L\left( {v,R_{s} ,k\left| D \right.} \right) = \prod\limits_{i}^{n} {p\left( {r_{i} \left| v \right.,R_{s} ,k} \right),}$$

where ${r}_{i}$ denotes the observed number of secondary cases arising from primary case $i$ and *p*(.) represents the probability mass function of the negative binomial distribution. We minimized the negative log-likelihood of Eq. (1). The 95% confidence intervals (CIs) of these parameters were obtained from profile likelihood.

As an alternative scenario, we assumed an exponential decrease in the reproduction number as a function of calendar time and ${R}_{t=0}$, the reproduction number of symptomatic cases at calendar time of zero, was estimated [23]. In this scenario, we assumed the time of transmission for primary symptomatic and asymptomatic cases was the time of illness onset and the time of exposure, respectively. To explore the potential role of symptomatic transmission in producing symptomatic secondary infections, we assumed that the number of symptomatic secondary cases among all secondary cases followed a binomial distribution with parameters *p* and *q*, representing the proportions of secondary symptomatic cases produced by primary symptomatic and asymptomatic cases, respectively. We estimated these parameters jointly with $R_{s}$, $v$ and $k$.

We also estimated parameters governing the probability distribution function of transmissibility relative to illness onset. According to He et al. [24], we assumed that the probability followed a gamma distribution. The probability distribution function of the serial interval, $s\left(\tau \right)$, can be then modeled by convolution as:

2 $$s\left(\tau \right)={\int }_{-m}^{\tau }h\left(\sigma \right)f\left(\tau -\sigma \right)d\sigma ,$$

where $h\left(\tau \right)$ and $f(\tau )$ are probability density functions of the relative frequencies of secondary transmission with respect to time since illness onset and the incubation period, respectively, and $m$ represents the start of infectiousness relative to illness onset. For $f(\tau )$, we used a lognormal distribution with a mean of 5.2 days, estimated using data from 425 patients in Wuhan, China [25]. The value of $m$ was assumed as 6 days based on the shortest observed serial interval in our transmission network. Given the total of *w* observations of serial intervals for secondary cases *j*, the likelihood of observing the serial intervals ${\tau }_{j}$ can be written as:

3 $$L\left( {\theta \left| D \right.} \right) = \prod\limits_{j}^{w} {s\left( {\tau_{j} \left| \theta \right.} \right),}$$

where $\theta$ is the vector of the parameters (e.g., the shape and rate parameters) of the gamma distribution. In total, *w* = 18 pairs of symptomatic primary cases and secondary cases with dates of illness onset available were included in the analysis. Finally, to complement our investigation of the transmissibility of asymptomatic cases, we explored the impact of isolation on these estimates using the probability distribution function of the serial interval shortened by isolation [23]. We formulated a parameter, ε denoting the relative risk of secondary transmission among isolated individuals, similarly to a previous study [23]. We jointly estimated $\varepsilon$ with $R_{s}$, $v$ and $k$ (see Supplementary Information). All statistical data were analyzed using R version 4.0.3 [26].

### Ethical considerations

This study analyzed data that are publicly available. The datasets used in our study were de-identified and fully anonymized in advance. The analysis of publicly available data without identity information did not require ethical approval.

## Results

**Fig. 2:**
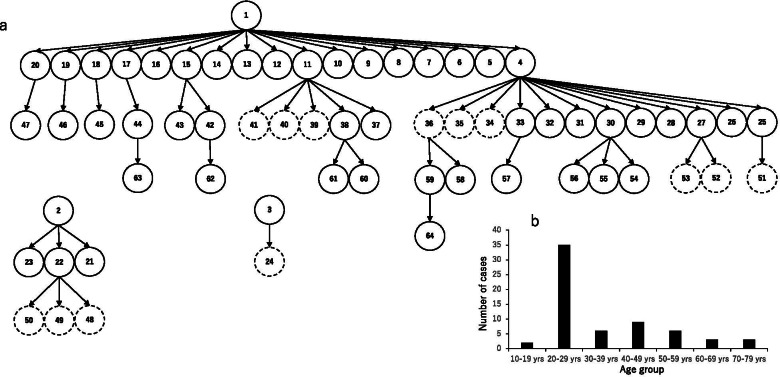
Chains of transmission and age distribution of cases in a cluster of SARS-CoV-2 infections among university students in Kyoto, Japan. **a** Transmission networks within the cluster. Solid and dotted circles indicate symptomatic and asymptomatic cases, respectively. **b** Age distribution of cases in the cluster

On the basis of contact tracing data for 74 cases, we reconstructed the transmission networks of 64 cases (51 symptomatic cases and 13 asymptomatic cases). For the remaining 10 cases, we were not able to identify transmission events, so we excluded them from subsequent analyses (Fig. 2a). Most infections (55%) occurred in individuals aged 20–29 years (Fig. 2b). The network comprised a total of five generations, with two symptomatic cases acting as “super-spreaders” and giving rise to more than 10 secondary infections (Case 1 and Case 4 in Fig. 2a).

Using the negative binomial distribution, $R_{s}$, the reproduction number for a symptomatic primary case, was estimated at 1.14 (95% CI: 0.61–2.09). $v$, the relative reproduction number for asymptomatic cases, was estimated at 0.19 (95% CI: 0.03–0.66), indicating that the reproduction number of asymptomatic primary cases was insufficient to maintain chains of transmission. The dispersion parameter of the negative binomial distribution was estimated at 0.24 (95% CI: 0.13–0.47) (Table 1).

**Table 1:** Epidemiological parameters of SARS-CoV-2 transmission using two models

	Base case model	Exponential decrease model
Parameters	Maximum likelihood estimate (95% CI)	Maximum likelihood estimate (95% CI)
*R*_s_ or *R*_t=0_	1.14 (0.61–2.09)	12.6 (0.69–37.0)
*v*	0.19 (0.03–0.66)	0.07 (0.01–0.79)
*k*	0.24 (0.13–0.47)	0.26 (0.11–0.63)
*δ*	-	0.29 (0.10–0.61)

*CI* confidence interval. Parameters included the reproduction number of symptomatic cases (*R*_s_), the reproduction number of symptomatic cases at calendar time zero (*R*_t=0_), the reproduction number for an asymptomatic case with respective to a symptomatic case (*v*), the dispersion parameter (*k*), and the exponentially decreasing rate of secondary transmission (*δ*)

The 95% CI of $v$ was relatively broad. Thus, to assess the uncertainty of $v$, we estimated $R_{s}$ and $v$ given fixed dispersion parameters of 0.05, 0.1, 0.2, 0.3, 1, or 10. The range of 0.05–0.2 was consistent with the 95% credible interval of the dispersion parameter estimated in the World Health Organization situation report [20], while dispersion parameters of 1 and 10 implied that the numbers of secondary cases per primary symptomatic case approximately follow a geometric or Poisson distribution, respectively. The 95% CI of $v$ narrowed as dispersion parameters increased, with a 95% CI of 0.06–0.5 at k = 10 (Table 2). Under a scenario where the reproduction number exponentially decreases as a function of calendar date, ${R}_{t=0}$ and $v$ were estimated at 12.6 (95% CI: 0.69–37.0) and 0.07 (95% CI: 0.01–0.79), respectively (Table 1). The value of $v$ was not precisely calculable when jointly estimated with the proportion of symptomatic cases; when $v$ was fixed as 0.2, the proportion of symptomatic cases arising from each primary symptomatic case and asymptomatic case was estimated at 0.82 (95% CI: 0.49–0.95) and 0.78 (95% CI: 0.64–0.87), respectively.

**Table 2:** Sensitivity of the reproduction number (*R*_s_) and relative transmissibility among asymptomatic individuals (*v*) to assumed values of the dispersion parameter, *k*

	*R*_s_	*v*
Dispersion parameter, *k*	95% CI	95% CI
0.05	0.31–3.78	0.02–0.90
0.10	0.45–2.76	0.02–0.79
0.20	0.58–2.20	0.03–0.68
0.30	0.64–1.99	0.04–0.63
1.00	0.78–1.66	0.05–0.54
10.00	0.87–1.50	0.06–0.50

*CI* confidence interval

Assuming that infectiousness started 6 days prior to illness onset and that the probability density function of secondary transmission relative to illness onset followed a gamma distribution, the shape and scale parameters were estimated at 5.49 (95% CI: 1.99–14.74) and 1.00 (95% CI: 0.35–2.57), respectively. The model using the probability density function of the serial interval shortened by case isolation did not converge to allow an explicit estimation of $\varepsilon$, the isolation effect, although the estimate was close to the lower bound. ${R}_{s}$, $v$ and $k$ were estimated at 1.58 (95% CI: 0.68–3.47), 0.32 (95% CI: 0.02–0.95) and 0.26 (95% CI: 0.12–0.57), respectively (see Supplementary Table 1). Even when assuming that the relative frequency of secondary transmission with respect to time since illness onset was known and fixed using the estimates of He et al. [15], a stable estimate of $\varepsilon$ was not successfully obtained (data not shown).

## Discussion

We assessed the relative transmissibility of asymptomatic SARS-CoV-2 infections in terms of the reproduction number and the serial dependence of symptomatic infection using cluster data and chains of transmission during the early stages of the epidemic in Kyoto, Japan. Assuming that the distribution of secondary cases followed a negative binominal distribution, we estimated the reproduction number of symptomatic cases as 1.14 (95% CI: 0.61–2.09) and the relative reproduction number for asymptomatic cases as 0.19 (95% CI: 0.03–0.66), respectively. ${R}_{t=0}$ (= 12.6) was much larger than ${R}_{s}$ because the former is the reproduction number at the biggening of the epidemic calendar time, with assumption of an exponential decrease over the course of epidemic, while the latter was assumed to be a constant throughout the course of epidemic. There was no apparent increased tendency for symptomatic primary case to produce symptomatic secondary cases. Because movement of all identified close contacts was restricted for 14 days, we also assessed the relative transmissibility in the model using the probability density function of the generation interval adjusted for the isolation period. Unfortunately, joint estimation of the effectiveness of case isolation with other parameters was unsuccessful.

Two published studies [15, 16] reported reproduction number estimates for asymptomatic cases of SARS-CoV-2 infection (Table 3). Using data on contact tracing from a first generation of 191 cases (161 symptomatic and 30 asymptomatic cases) during the very early stages of the epidemic in Ningbo, China, He et al. [15] reported that the reproduction numbers of symptomatic and asymptomatic cases were 0.78 and 0.20, respectively, indicating a risk ratio for transmission of 0.26 in asymptomatic cases. Our point estimate of the relative reproduction number for asymptomatic cases (0.19) was consistent with this finding. Recently, we reported that the relative transmissibility of asymptomatic cases was 0.27 by analyzing transmission networks within an early cluster in Tokyo and Kanagawa [16]; this result was also broadly consistent with the findings of the current study. Two other contact tracing studies assessed the transmissibility of asymptomatic infections using epidemiological measurements other than the reproduction number. Using the incidence rate ratio adjusted for age, sex and serological status, a recent report from Singapore found that the relative transmissibility of asymptomatic cases was around one-third (0.26) that of symptomatic cases [13], again agreeing with our results. Of note, this result was based on regular screening of workers in specific industries, not intensive investigations triggered by notification of clusters such as in our study. By contrast, a study in Brunei investigating an outbreak followed by a cluster at a religious event showed that the secondary attack rate ratio for asymptomatic vs. symptomatic cases (including pre-symptomatic cases) was close to parity (1.1; calculated from data in Supplementary Information) [14]. However, when the analysis was restricted to cases in households, the attack rate ratio was 0.37. A household transmission study in Japan indicated that the secondary attack risk of asymptomatic primary cases was 11.8% while overall secondary attack risk was 19.0% [27]. These results suggest that the transmissibility of asymptomatic cases is less than half that of symptomatic cases.

**Table 3:** Summary of transmission profiles of asymptomatic cases in contact tracing studies

Study	Setting	Sample size	Measurement of relative infectivity of asymptomatic cases
He et al. [15]	Ningbo city, China	52 asymptomatic and 271 symptomatic cases	Ratio of reproduction numbers: 0.26^b^
Nakajo et al. [16]	Tokyo and Kanagawa, Japan	12 asymptomatic and 24 symptomatic cases	Ratio of reproduction numbers: 0.27 (95% CI: 0.03–0.81)
Sayampanathan et al. [13]	Singapore	3035 contacts of asymptomatic cases and 755 contacts of symptomatic cases	Incidence rate ratio: 0.26^b^
Chaw et al. [14]	Brunei	106 contacts of asymptomatic cases and 1595 contacts of symptomatic cases^a^	Attack rate ratio: 1.12^c^

^a^ pre-symptomatic cases were counted as symptomatic cases

^b^ reciprocal of reported values

^c^ calculated manually from Supplementary Table 1

Our findings help to critically assess the value of contact tracing for COVID-19, including the cluster-based approach [19], in preventing major epidemics. In addition, this approach would enable us to identify contact history even for asymptomatic cases in a back-ward manner and further to elucidate the transmission profile for them. It is, however, typically not feasible to identify all infected individuals, and the impetus for contact tracing is usually notification of laboratory confirmed symptomatic cases. There are two explanations for this. First, even if an initially asymptomatic index case were missed (untraced), the reproduction number was estimated as *R*_a_ = 0.21, substantially below parity, and the resulting outbreak would be very likely to decline to extinction. Second, supposing that only a proportion *x* of contacts are traced, which may be correlated with 1- the asymptomatic ratio, the reproduction number with contact tracing would be (1-*x*)((1-*z*)*R*_s_ + *zR*_a_) + *ux*(*yR*_s_ + (1-*y*)*R*_a_), where *z* is the asymptomatic ratio, *y* is the proportion of traced asymptomatic contacts that would eventually develop symptoms, and *u* is the reduction factor resulting from contact tracing. Assuming that *u*≈0 and *x* = *kz*, where *k* is a constant, the reproduction number with contact tracing can be simplified to (1-*kz*)((1-*z*)*R*_s_ + *zR*_a_). Assuming that *k* = 1, *z* is 0.30 or 0.50 [2] and *R*_s_ = 1.14, the resulting reproduction number is 0.60 and 0.34, respectively. Thus, when tracing capacity is substantial, it is justified to implement contact tracing beginning with symptomatic cases, especially if testing capacity is limited.

Our exploratory analysis suggested that a symptomatic case does not have a higher tendency to produce additional symptomatic infections than asymptomatic cases. Unfortunately, there was also no suggestion that asymptomatic secondary cases were more likely to be generated from an asymptomatic primary case. This finding supports the use of a classical branching process model of the generation-dependent transmission process in an independent manner.

This study has several limitations. First, the sample size was small, involving a broad uncertainty bound and a wide 95% CI. The number of asymptomatic cases was 13, accounting for approximately 20% out of all cases, and the number of primary asymptomatic case was only one in this cluster. So, we would need a larger number of asymptomatic cases to obtain more precise estimates of the relative transmissibility and effectiveness of case isolation. Second, we had to exclude 10 symptomatic cases in the cluster because we could not identify specific transmission events. Three cases among these 10 were possibly infected by asymptomatic individuals in the transmission network. However, in the hypothetical scenario that a single asymptomatic case infected all three cases, we estimated that the relative transmissibility for asymptomatic cases was 0.35. This figure is compatible with our baseline estimate. Third, we could not estimate the effect of the first 14 days of quarantine period on transmission profile, because the date of identification as being close contact for each subject was not available on our transmission network. Lastly, our study was of young and otherwise healthy individuals attending nighttime parties, so the generalizability of our findings to other populations is questionable (e.g., elderly adults with underlying comorbidities). We recently investigated a cluster of 36 cases originating from a nighttime party that was propagated to health-care facilities. The results were similar to those of the current study, suggesting that the transmissibility of asymptomatic cases may not be highly sensitive to setting [16]. However, this should be confirmed by additional studies via meticulous observational efforts.

Despite these limitations, we successfully estimated the relative transmissibility of asymptomatic cases of SARS-CoV-2 infection within a transmission network. The reduced estimate of the reproduction number of asymptomatic cases suggested that contract tracing focusing on symptomatic index cases may be justified when there is limited testing capacity.

## Conclusions

Using data on transmission networks during an early epidemic in Japan, we estimated the relative reproduction number of asymptomatic cases of SARS-CoV-2 infection as 0.22. There was no apparent tendency for symptomatic primary cases to preferentially produce symptomatic secondary cases. To extend these findings to other transmission settings, additional studies on the transmission potential of asymptomatic cases are necessary.

## Supplementary Information

**Additional file 1.**

**Additional file 2.**

**Additional file 3.**

## Data Availability

The original data used to construct the epidemic curve are available as Online Supporting Material.

## Abbreviations

CI: Confidence interval
COVID-19: Coronavirus disease 2019
SARS-CoV-2: Severe acute respiratory syndrome coronavirus-2
