# Supplementary material for: Exploring secondary SARS-CoV-2 transmission from asymptomatic cases using contact tracing data
Source: Theor Biol Med Model. 2021 Jul 16;18:12. doi: 10.1186/s12976-021-00144-z (PMC8284042; doi:10.1186/s12976-021-00144-z)
Supplement: Supplementary file 3 — Additional file 3. [file 12976_2021_144_MOESM3_ESM.docx]

# Supplementary Information

**Derivation of the probability density function of the serial interval shortened by isolation and expected number of secondary cases**

As a basis of this approach, we employed the renewal equation [1]:

| $j\left( t \right)=\int_{0}^{\infty} A(\tau)j\left( t-\tau\right)d\tau,$ | (1) |
| --- | --- |

where $j\left( t \right)$ is the incidence at calendar time $t$and $A\left( \tau\right)$ is the rate of secondary transmission per case at infection age $\tau$. Note that $g\left( \tau\right)$, the probability density function of the generation interval (i.e., the time from infection of a primary case to the infection of a secondary case by the primary case), is given by:

| $g\left( \tau\right)=\frac{A(\tau)}{\int_{0}^{\infty} A(x)dx}.$ | (2) |
| --- | --- |

We were interested in differences in the transmissibility profiles of symptomatic cases and asymptomatic cases. For a symptomatic individual, *𝜆*$\left( \tau\right)$, the probability density function of the interval from his/her illness onset to transmission to a secondary case was used to capture information on transmissibility relative to the time since illness onset, including before the time of illness onset. In our study, all cases were immediately isolated after laboratory confirmation, and thus we assumed that the transmissibility profile for each symptomatic case was altered following isolation. According to Chan and Nishiura [2], let us denote *𝜀* as the relative reduction in the rate of secondary transmission in an isolated individual. *𝜀* =0 indicates isolation has no effect on transmission profile. The probability density function of infectiousness relative to illness onset with isolation, *ℎ*$\left( \tau\right)$, will then be given by:

| $h\left( \tau\right)=\left\{ \begin{aligned} \frac{\lambda(\tau)}{\int_{-m}^{k} \lambda(s)ds+(1-\varepsilon)\int_{k}^{\infty} \lambda\left( s \right)ds} for \tau\leq k \\ \frac{(1-\varepsilon)\lambda(\tau)}{\int_{-m}^{k} \lambda(s)ds+(1-\varepsilon)\int_{k}^{\infty} \lambda\left( s \right)ds} for \tau>k \end{aligned} \right.,$ | (3) |
| --- | --- |

where $k$ and $m$ represent the time of isolation and the beginning of infectiousness relative to illness onset, respectively. Then, the probability density function of the observed serial interval will be given as a convolution of two probability density functions, $h\left( \tau\right)$ and $f(\tau)$; the latter is the probability density function of the incubation period,

| $s\left( \tau\right)=\int_{-m}^{\tau} h\left( \sigma\right)f\left( \tau-\sigma\right)d\sigma.$ | (4) |
| --- | --- |

$R_{s}$, the expected number of secondary cases generated by a primary symptomatic case under isolation, can be written as:

| $R_{s}=R_{0,s}\left( \int_{{-m}}^{x} \lambda\left( \tau\right)d\tau+\left( 1-\varepsilon\right)\int_{x}^{\infty} \lambda\left( \tau\right)d\tau\right),$ | (5) |
| --- | --- |

where $R_{0,s}$ indicates the basic reproduction number for a primary symptomatic case and *x* is the disease age (i.e., time since illness onset) when primary cases are isolated. For a primary asymptomatic case (i.e., a case who never manifested symptoms during the observation period), we substitute the probability density function of the generation interval, $g\left( \tau\right)$, for $\lambda\left( \tau\right)$ in Eq. (5). $g\left( \tau\right)$ is modeled as the convolution of two probability density functions, $\lambda\left( \tau\right)$ and $f(\tau)$, as:

| $g\left( \tau\right)=\int_{0}^{\tau} f\left( \sigma\right)\lambda\left( \tau-\sigma\right)d\sigma.$ | (6) |
| --- | --- |

Assuming the reduction of transmissibility resulting from isolation is the same as for symptomatic cases, $R_{a}$, the expected number of secondary cases generated by a primary asymptomatic case under isolation can be described as:

| $R_{a}=R_{0,a}\left( \int_{0}^{x} g\left( \tau\right)d\tau+\left( 1-\varepsilon\right)\int_{x}^{\infty} g\left( \tau\right)d\tau\right),$ | (7) |
| --- | --- |

where $R_{0,a}$ and $x$ indicate the basic reproduction number for a primary asymptomatic case and the time of isolation, respectively. Note that time zero in the integral range indicates the time of exposure. Let us denote $v$ as the relative reduction in the basic reproduction number for an asymptomatic case with respective to a symptomatic case. Then, equation (7) can be rewritten as:

| $R_{a}={vR}_{0,s}\left( \int_{0}^{l} g\left( \tau\right)d\tau+\left( 1-\varepsilon\right)\int_{l}^{\infty} g\left( \tau\right)d\tau\right),$ | (8) |
| --- | --- |

where $l$ indicates the time of isolation. The datasets used here were reported in a discrete time interval (days) so we discretized Eqs. (4), (5) and (8) as:

| $s_{\tau}=\sum_{s=1}^{m+\tau+1} h_{s+m+1}f_{\tau-s-m-1},$ | (9) |
| --- | --- |
| $R_{s}=R_{0,s}\left( \sum_{\tau=1}^{k+m+1} \lambda_{\tau}+(1-\varepsilon)\sum_{\tau=k+m+2}^{T} \lambda_{\tau} \right),$ | (10) |

and

| $R_{a}={vR}_{0,s}\left( \sum_{\tau=1}^{l+m+1} g_{\tau}+(1-\varepsilon)\sum_{\tau=l+m+2}^{T} g_{\tau} \right),$ | (11) |
| --- | --- |

where $h_{\tau}$, $f_{\tau}, \lambda_{\tau}$ and $g_{\tau}$ are the probability mass functions corresponding to $h\left( \tau\right), f\left( \tau\right), \lambda\left( \tau\right)$ and $g\left( \tau\right)$, respectively, and $g_{\tau}= \sum_{s=1}^{\tau-1} h_{\tau-s}\lambda_{s}$.

**Likelihood functions**

We used a maximum-likelihood method to estimate parameters. The likelihood function, $L(\theta|D)$, consists of two parts, the observed length of the serial interval, $L_{1}(\theta|D)$, and the observed number of secondary cases, $L_{2}(\theta|D)$:

| $L\left( \theta\vert D \right)=L_{1}\left( \theta\vert D \right)L_{2}\left( \theta\vert D \right).$ | (12) |
| --- | --- |

Let $D_{1}$ be the number of datasets of observed serial intervals. Then, the first likelihood is written as:

| $L_{1}\left( \varepsilon, \boldsymbol{\alpha} \vert D \right)= \prod_{i}^{D_{1}} s\left( t_{i} \vert\varepsilon,\boldsymbol{\alpha} \right),$ | (13) |
| --- | --- |

where $\boldsymbol{\alpha}$ is the vector of parameters for $\lambda\left( \tau\right)$ and $t_{i}$ is the observed serial interval. We assumed that $\lambda\left( \tau\right)$ follows a gamma distribution [3]. For $f(\tau)$, we used a lognormal distribution with a mean of 5.2 days estimated based on data from 425 patients in Wuhan [4]. The value of $m$ was set as 6 days based on the observed smallest serial interval in our transmission network. We assumed that the observed number of secondary cases derived from either a primary symptomatic or asymptomatic case will follow a negative binomial distribution with $R_{s}$ or $R_{a}$ as the mean, respectively. Let $D_{2}$ be the number of datasets for observed secondary cases. Then, we can describe the second likelihood as:

| $L_{2}\left( \varepsilon, v, R_{0,s}\boldsymbol{, \alpha,}k \vert D \right)= \prod_{i}^{D_{2}} p\left( r_{i} \vert\varepsilon,v,R_{0,s}\boldsymbol{, \alpha,}k \right)$  $=\prod_{i}^{D_{2}} NB\left( r_{i}; k, \frac{k}{R_{-}+k} \right),$ | (14) |
| --- | --- |

where $R_{-}$ is $R_{s}$or $R_{a}$, $k$ is the dispersion parameter for the offspring distribution and $r_{i}$ is the observed number of secondary cases arising from primary case $i$. As in the main analysis, we adopted two assumptions for $R_{0,s}$ (i.e., taking a constant value throughout the course of the epidemic vs. the value exponential decreases as a function of calendar time). In the latter scenario where ${R\left( t \right)=R}_{0,s}e^{-\delta t}$, $\delta$, the exponential rate, was jointly estimated with the initial reproduction number $R_{0,s}$. We minimized the negative log-likelihood of Eq. (12). The 95% CIs for the parameters were obtained by profile likelihood. The estimates are shown in Supplementary Table 1.

# References

1. Nishiura H, Chowell G. The effective reproduction number as a prelude to statistical estimation of time-dependent epidemic trends. In: Chowell G, Hayman JM, Bettencourt LMA, Castillo-Chavez C, editors. Mathematical and statistical estimation approaches in epidemiology. Dordrecht: Springer; 2009. pp. 103–21.

2. Chan YH, Nishiura H. Estimating the protective effect of case isolation with transmission tree reconstruction during the Ebola outbreak in Nigeria, 2014. J R Soc Interface. 2020;17(169):20200498.

3. He X, Lau EHY, Wu P, Deng X, Wang J, Hao X, et al. Temporal dynamics in viral shedding and transmissibility of COVID-19. Nat Med. 2020;26(5):672–5.

4. Li Q, Guan X, Wu P, Wang X, Zhou L, Tong Y, et al. Early transmission dynamics in Wuhan, China, of novel coronavirus-infected pneumonia. N Engl J Med. 2020;382(13):1199–207.

**Supplementary Table 1. Parameters estimated in the two models.**

|  | Base case | Exponential decrease |
| --- | --- | --- |
| Parameters | Mean (95% CI) | Mean (95% CI) |
| *R*_s_ or *R*_t=0_ | 1.58 (0.68–3.47) | 8.05 (1.11–17.7) |
| *ɛ* | 0.001 (0.001–NaN) | 0.001(0.001–0.999) |
| *v* | 0.32 (0.02–0.95) | 0.15 (0.02–0.75) |
| *k* | 0.26 (0.12–0.57) | 0.43 (0.17–1.06) |
| *α*_1_ | 5.49 (1.17–9.19) | 5.49 (1.96–14.4) |
| *α*_2_ | 1.00 (0.35–2.57) | 1.00 (0.35–2.58) |
| *δ* | - | 0.23 (0.10–0.46) |

CI: confidence interval; NaN, not a number.

The included parameters were the reproduction number of symptomatic cases (*R*_s_), the reproduction number of symptomatic cases at calendar time of zero (*R*_t=0_), the relative reduction in the rate of secondary transmission in an isolated individual (*ɛ*), the relative reduction in the reproduction number for an asymptomatic case with respect to a symptomatic case (*v*), the dispersion parameter (*k*), the shape (*α*_1_) and rate (*α*_2_) parameter of the gamma distribution, and the exponentially decreasing rate of secondary transmission (*δ*).
